# Effect of blood pressure on recovery outcomes in inpatients with atherothrombotic infarction: A retrospective cross-sectional study

**DOI:** 10.1097/MD.0000000000041796

**Published:** 2025-03-14

**Authors:** Naohito Saito, Tetsuo Nishikawa, Tetsuo Ota

**Affiliations:** a Department of Physical Medicine and Rehabilitation, Sunagawa City Medical Center, Sunagawa, Hokkaido, Japan; b Endocrinology & Diabetes Center, Yokohama Rosai Hospital, Yokohama, Kanagawa, Japan; c Department of Physical Medicine and Rehabilitation, Asahikawa Medical University Hospital, Asahikawa, Hokkaido, Japan.

**Keywords:** activities of daily living, atherothrombotic infarction, Barthel index, blood pressure, rehabilitation

## Abstract

Stroke is one of the leading causes of death worldwide, and the relationship between blood pressure (BP) and outcomes after atherothrombotic infarction has been studied from various perspectives. However, the relationship of BP with rehabilitation outcomes and activities of daily living after atherothrombotic infarction has not been studied extensively. This study aims to investigate the effect of BP on rehabilitation outcomes and activities of daily living after atherothrombotic infarction. In this retrospective cross-sectional study, we analyzed data obtained from the Japan Association of Rehabilitation Database for inpatients undergoing rehabilitation using Wilcoxon’s test, Pearson’s chi-squared test, or Fisher’s exact test. The patients were initially categorized into hypertensive and non-hypertensive groups and were assessed using the 10-item Barthel index (BI) activities and total BI at hospital discharge. The patients were further dichotomized into dependent (patients with scores of 0 for each activity) and nondependent groups based on the BI activities. Compared with hypertensive conditions (n = 108), non-hypertensive conditions (n = 213) were associated with higher dependence (feeding, transfers, toilet use, dressing, and bowel and bladder control). The proportion of non-hypertensive patients with a severely low BI (0–15) was higher than that of hypertensive patients. The hypertensive group had a greater increase in the BI (25 vs 15, *P* = .006) and daily BI (1.2 vs 0.74, *P* = .014) than the non-hypertensive group. During in-hospital rehabilitation, hypertensive patients recovered more efficiently than their non-hypertensive counterparts, emphasizing the need for personalized rehabilitation plans based on their individual BP profiles. Our results underscore the impact of BP on inpatients after atherothrombotic infarction, indicating that more non-hypertensive inpatients are affected by BP while receiving treatment than are hypertensive inpatients during rehabilitation.

## 1. Introduction

In 2019, stroke was the second leading cause of death and the third leading cause of death and disability combined worldwide,^[1]^ with approximately 12.2 million incident cases of stroke, 101 million prevalent cases of stroke, 143 million disability-adjusted life-years due to stroke, and 6.55 million deaths due to stroke reported.

The effect of blood pressure (BP) on ischemic stroke has been investigated in several aspects, including primary prevention, use of tissue plasminogen activators in the acute phase, secondary prevention, BP variability, and choice of antihypertensive medication.^[2,3]^ Previous studies have reported that high BP is closely associated with stroke and that its reduction could decrease the incidence of stroke.^[4]^ Additionally, other studies have reported that both low and high BPs are associated with poor functional outcomes in acute ischemic stroke.^[5–9]^ Severe outcomes in terms of activities of daily living (ADL) are observed during rehabilitation in some inpatients with low BP, and most patients with orthostatic hypotension (OH) are asymptomatic or have few nonspecific symptoms, thus accounting for a high number of unrecognized cases.^[10]^ The association between baseline OH and the risk of falls has been reported.^[11]^ OH management can be challenging, despite multiple pharmaceutical and non-pharmaceutical options available, which have adverse effects.^[12,13]^ Although the effect of high BP on stroke has been widely studied, the effects of low BP have gained less attention, and the relationship of BP with rehabilitation outcomes and ADL after atherothrombotic infarction has not been extensively studied.

Therefore, this study aimed to analyze the effect of BP on outcomes at hospital discharge in patients with atherothrombotic infarction from the Japan poststroke rehabilitation database using the Barthel index (BI).^[14,15]^

## 2. Methods

### 2.1. Data source

**Figure 1.**
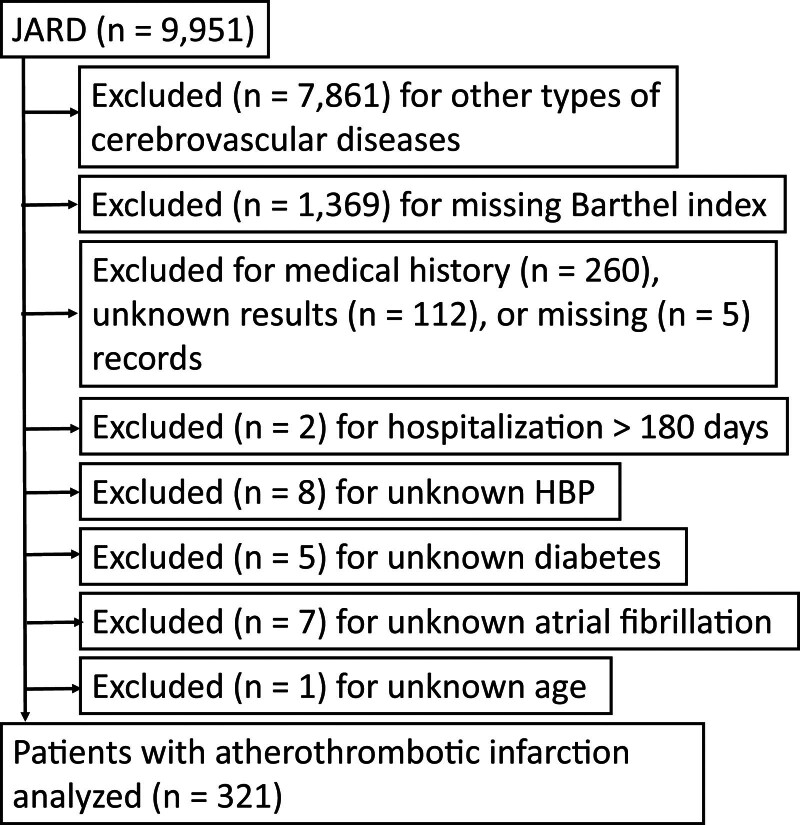
Flowchart of the selection of inpatients with atherothrombotic infarction. HBP = high blood pressure (hypertension), JARD = Japan Association of Rehabilitation Database.

In this retrospective cross-sectional study, we analyzed data from the Japan Association of Rehabilitation Database (obtained from http://square.umin.ac.jp/JARD/index.html), which were collected from inpatients with atherothrombotic infarction. This study was conducted according to the principles of the Declaration of Helsinki, and the study protocol was reviewed and approved by the Sunagawa City Hospital Ethical Committee (approval number: #2019-35). We analyzed a deidentified dataset in this study; therefore, the requirement for obtaining written informed consent from the patients was waived. The JARD contains data on inpatients with most types of cerebrovascular disease (CVD) (e.g., lacunar infarction, atherothrombotic infarction, cardioembolic infarction, and other types of infarctions, as well as hypertensive hemorrhage, subarachnoid hemorrhage, and other types of hemorrhages), femoral neck fracture,^[16]^ spinal cord injury, and/or various other diseases or injuries. We initially identified patients admitted to the rehabilitation ward or conventional ward who were observed until discharge and voluntarily registered in the JARD at various hospitals in Japan between January 2006 and December 2013. For the present study, we focused on inpatients admitted to the conventional ward with a diagnosis of atherothrombotic infarction. Before statistical analysis, we excluded inpatients with CVDs other than atherothrombotic infarction (Fig. 1), missing records of the BI at hospital discharge or admission, past CVDs, unknown or missing records of CVDs, hospital stay > 180 days (rehabilitation in our country is limited to 180 days after admission as paresis improves within 180 days after a stroke), and missing records of hypertension, diabetes, atrial fibrillation, or age.

### 2.2. Hypertension, diabetes, and atrial fibrillation

Hypertension, diabetes, and atrial fibrillation were diagnosed and categorized by medical workers at each hospital. According to the general definition used in Japan, hypertension was defined as 2 consecutive measurements of systolic BP (SBP) ≥ 140 mm Hg with or without diastolic BP ≥ 90 mm Hg. Patients using antihypertensive medications were also categorized as having hypertension, regardless of their BP measurements. Diabetes was diagnosed either based on the diagnostic criteria of the Japan Diabetes Society in the chronic stage or based on patients’ medical history.^[17]^

### 2.3. Brunnstrom recovery stage

Given the involvement of paresis in ADL deterioration among patients after stroke,^[18–20]^ we assessed paresis using the Brunnstrom recovery stage (BRS), which is an evaluation method that divides the recovery stage of motor function in the upper limbs, hands and fingers, and lower limbs into 6 stages: stage 1, flaccid paralysis; stage 2, movements in a synergic pattern and emergence of spasticity; stage 3, voluntary synergic movements across joints and increased spasticity; stage 4, voluntary movements outside synergic patterns and decreasing spasticity; stage 5, control of individual or isolated movements; and stage 6, return to near-normal motor control. Further, the BRS was dichotomized at stages 1 (BRS 1), 2 (BRS 1 and 2), 3 (BRS 1, 2, and 3), 4 (BRS 1, 2, 3, and 4), or 5 (BRS 1, 2, 3, 4, and 5) to compare each category and its respective counterpart (Table 1).

**Table 1:** Patient characteristics.

	Dichotomization	Non-HBP (n = 108)	HBP (n = 213)	*P*-value
Female, n (%)	Female vs male	45 (42)	83 (39)	.64
Age, years, median (IQR)		70.5 (63–82)	71 (63–78)	.35
Hospital stay, days, median (IQR)		20 (13–30)	20 (13–33)	.52
Atrial fibrillation, n (%)		5 (5)	10 (5)	.97
Diabetes, n (%)		32 (30)	87 (41)	.05*
BRS-UE		88	181	
1, n (%)	1 vs 2–6	5 (6)	3 (2)	.11
1 and 2, n (%)	1–2 vs 3–6	15 (17)	21 (12)	.21
1, 2, and 3, n (%)	1–3 vs 4–6	24 (27)	35 (19)	.14
1, 2, 3, and 4, n (%)	1–4 vs 5–6	27 (31)	45 (25)	.31
1, 2, 3, 4, and 5, n (%)	1–5 vs 6	44 (50)	69 (38)	.06
BRS-HF		88	181	
1, n (%)	1 vs 2–6	5 (6)	6 (3)	.35
1 and 2, n (%)	1–2 vs 3–6	17 (19)	20 (11)	.06
1, 2, and 3, n (%)	1–3 vs 4–6	21 (24)	36 (20)	.45
1, 2, 3, and 4, n (%)	1–4 vs 5–6	27 (31)	44 (24)	.26
1, 2, 3, 4, and 5, n (%)	1–5 vs 6	41 (47)	73 (40)	.32
BRS-LE		88	179	
1, n (%)	1 vs 2–6	5 (6)	4 (2)	.16
1 and 2, n (%)	1–2 vs 3–6	11 (13)	12 (7)	.11
1, 2, and 3, n (%)	1–3 vs 4–6	16 (18)	23 (13)	.24
1, 2, 3, and 4, n (%)	1–4 vs 5–6	24 (27)	37 (21)	.22
1, 2, 3, 4, and 5, n (%)	1–5 vs 6	38 (43)	64 (36)	.24

*Statistically significant.

BRS = Brunnstrom Recovery Stage, HBP = high blood pressure (hypertension), HF = hand and finger, IQR = interquartile range, LE = lower extremity, UE = upper extremity.

### 2.4. Barthel index

**Figure 2.**
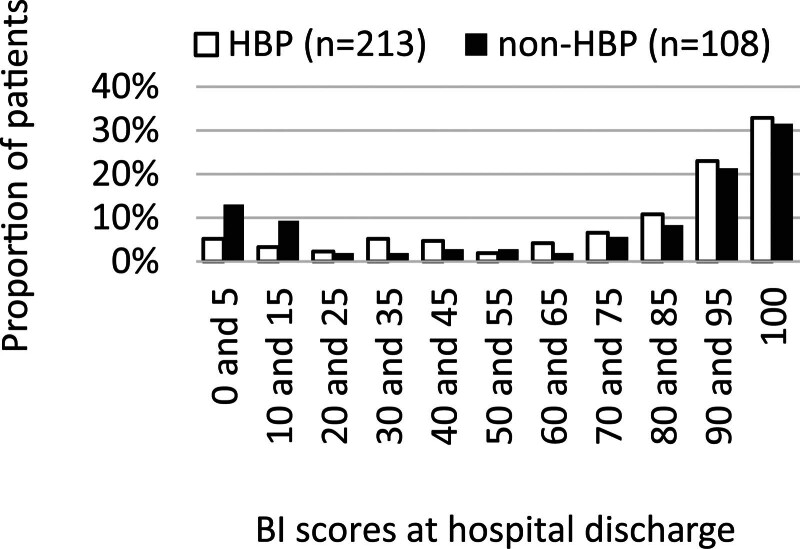
Proportion of hypertensive (n = 213) and non-hypertensive (n = 108) patients and related BI at hospital discharge. BI = Barthel index, HBP = high blood pressure (hypertension).

ADLs were assessed using the BI at hospital admission and discharge.^[15]^ The BI is a simple index of independence that measures the ability of patients with neuromuscular or musculoskeletal disorders to care for themselves and is known to have excellent inter-rater reliability for standard administration after stroke.^[21]^ The BI evaluates 10 basic activities of self-care (feeding, grooming, dressing, toilet use, bathing, control of bowels, and bladder control) and mobility (transfers, walking on level surfaces, and stair climbing), with a total score ranging from 0 (totally dependent) to 100 (totally independent). Specifically, a score of 0 for each activity indicates dependence and the need for assistance. We categorized the study population into dependent activity (scores of 0 for each activity) and nondependent activity groups based on their BI (Table 2). The total BI at hospital discharge was stratified for visual comparison between hypertensive and non-hypertensive inpatients (Fig. 2). The BI for each patient at discharge was subtracted from that at admission to calculate the BI increase. Each patient’s BI increase was divided by the number of days of hospitalization to assess the daily BI increase.

**Table 2:** Effect of hypertension on the outcomes of inpatients with atherothrombotic infarction after stroke rehabilitation.

BI	Dichotomization	Non-HBP (n = 108)	HBP (n = 213)	*P*-value
Feeding, n (%)	0 vs 5 and 10	13 (12)	9 (4)	<.001*
Transfers, n (%)	0 vs 5, 10, and 15	17 (16)	11 (5)	.001*
Grooming, n (%)	0 vs 5	35 (32)	49 (23)	.07
Toilet use, n (%)	0 vs 5 and 10	24 (22)	26 (12)	.02*
Bathing, n (%)	0 vs 5	63 (58)	120 (56)	.73
Walking, n (%)	0 vs 5, 10, and 15	24 (22)	31 (15)	.08
Stairs, n (%)	0 vs 5 and 10	42 (39)	64 (30)	.11
Dressing, n (%)	0 vs 5 and 10	28 (26)	28 (13)	.004*
Bowel control, n (%)	0 vs 5 and 10	25 (23)	27 (13)	.016*
Bladder control, n (%)	0 vs 5 and 10	24 (22)	27 (13)	.03*
BI score at discharge, median (IQR)		90 (31–100)	90 (65–100)	.19
BI score at admission, median (IQR)		52.5 (5–80)	50 (17–75)	.89
BI score increase, median (IQR)		15 (1.2–35)	25 (10–42)	.006*
Daily BI score increase, median (IQR)		0.74 (0.03–1.7)	1.2 (0.37–2.2)	.014*

*Statistically significant.

BI = Barthel Index, HBP = high blood pressure (hypertension), IQR = interquartile range.

### 2.5. Statistical analyses

Data were first tested for normality using the Shapiro–Wilk test. Parametric and nonparametric evaluations were used as appropriate. Data on patient age, BI, BI increase, daily BI increase, and length of hospitalization were compared using Wilcoxon’s test. Data on patient sex, BRS, and BI activities were compared using Pearson’s chi-squared test or Fisher’s exact test with a 2 × 2 contingency table. All statistical analyses were performed using the JMP16 software (SAS Institute, Inc., Cary, NC). *P*-values of < .05 were considered statistically significant.

## 3. Results

### 3.1. Data selection

Figure 1 shows the data selection procedure of this study. The JARD included data from 9951 inpatients with stroke from conventional wards; among them, 7861 inpatients were excluded because they had other CVDs. Patients with missing records of the BI (n = 1369, of which n = 1364 had missing records at hospital discharge and n = 5 at hospital admission) were also excluded. Furthermore, 400 patients were excluded because they were presumed to be outliers, had missing records, or had unknown results. These patients included those with past CVDs (n = 260), unknown results (n = 112), or missing records (n = 5) regarding the medical history of CVDs; those who were hospitalized for > 180 days (n = 2); and those with missing records of hypertension (n = 8), diabetes (n = 5), atrial fibrillation (n = 7), or age (n = 1). Finally, 321 patients (3.22%, 128 [40%] women) were enrolled and considered for the statistical analysis, among whom 213 were hypertensive (83 [39%] women) and 108 were non-hypertensive (45 [42%] women).

### 3.2. Patient characteristics

No significant differences were observed in sex (*P* = .64), age (*P* = .35), hospital stay (*P* = .52), or atrial fibrillation (*P* = .97) between the hypertensive and non-hypertensive groups (Table 1). The hypertensive group included more patients with a history of diabetes than the non-hypertensive group (87 [41%] vs 32 [30%]; *P* = .049). Furthermore, analysis of available paresis data from inpatients who experienced atherothrombotic infarction revealed no significant difference in the paresis of the upper limbs, hands and fingers, or lower limbs.

### 3.3. Activities of daily living

There was no significant difference in the total BI at admission (50 vs 52.5, *P* = .89) or the BI at discharge (90 vs 90, *P* = .19) between the hypertensive and non-hypertensive groups, although the interquartile ranges of the BI at discharge showed a slight difference (65 and 100 vs 31 and 100, respectively). The BI increase (25 vs 15, *P* = .006) and daily BI increase (1.2 vs 0.74, *P* = .014) during hospitalization were greater for the hypertensive group than for the non-hypertensive group. An increase in the proportion of non-hypertensive patients with a severely low BI (0–15) was observed (*P* < .001) after stratification of the BI in 10-unit increments (Fig. 2). The proportion of patients who required assistance at hospital discharge was significantly higher in the non-hypertensive group than in the hypertensive group (Table 2): feeding [13 (12) vs 9 (4), *P* = .0089], transfers [17 (16) vs 11 (5), *P* = .0015], toilet use [24 (22) vs 26 (12), *P* = .019], dressing [28 (26) vs 28 (13), *P* = .004], and control of bowels [25 (23) vs 27 (13), *P* = .016] and bladder [24 (22) vs 27 (13), *P* = .027].

## 4. Discussion

Age and paresis are known to affect the outcomes of patients with stroke.^[20]^ In this study, we determined the total BI and individual BI activities to investigate the impact of BP on inpatient ADL after atherothrombotic infarction and compared data of hypertensive and non-hypertensive patients undergoing BP treatment who showed no significant differences in age and paresis. We found that BP influences rehabilitation outcomes after atherothrombotic infarction and that hypertension positively influences ADL recovery, as there were improvements in several BI activities and changes in the BI, daily BI, and distribution of BI. Furthermore, our results suggest that some patients stratified into the non-hypertensive group might have difficulty in maintaining an optimal BP during sitting, standing, and walking training because of comorbidities such as low BP and OH at baseline or induced by stroke. We consider that this hypothesis might be true in the case of other types of strokes, such as cardioembolic and idiopathic infarctions, which we observed in our JARD dataset (data not shown). However, this is not necessarily true in the case of intracranial hemorrhages, such as subarachnoid hemorrhage, as hypertensive inpatients with subarachnoid hemorrhage were previously reported to have longer hospital stays and lower ADL at hospital discharge than non-hypertensive inpatients.^[22]^

Sink et al^[23]^ examined data from 9361 adults aged ≥ 50 years with SBP ranging between 130 and 180 mm Hg for the SBP Intervention Trial. They found that intensive BP control was associated with a greater risk of serious adverse events involving hypotension and possibly syncope but not falls and that OH measured at baseline was a risk factor for future serious adverse events, such as falls but not syncope or hypotension. Juraschek et al^[11]^ performed a prospective analysis of the association between baseline OH and risk of falls in the Atherosclerosis Risk in Communities Study. In a community-based, middle-aged population, OH was reported as an independent risk factor for falls in over 2 decades of follow-up; in particular, changes in diastolic BP were more strongly and significantly associated with a risk of falls than changes in SBP. In the present study, some BI activities, such as bathing, climbing stairs, and walking, were not significantly different between the 2 groups because they are relatively difficult compared to the other BI activities and were probably challenging for patients with atherothrombotic infarction.^[24]^ Other BI activities such as feeding, transfers, toilet use, and dressing are easier to perform than bathing, climbing stairs, and walking. However, they may be impossible to perform if patients cannot sit in bed because of low BP or, more importantly, remain in the standing posture.

Studies on the effect of BP on stroke have been reported by many groups using various methods^[6–9]^; however, reports on low BP and OH are limited. Phipps et al^[25]^ examined 60 patients with stroke and found that 16 (27%) patients had OH. Among those with OH, half were hypertensive, 7 were normotensive, and 1 was hypotensive. Leonardi-Bee et al^[5]^ reported that relatively low BP (SBP < 120 mm Hg), although an uncommon clinical finding (nearly 5% of patients), is also associated with poor outcomes, considering that low BP has been found to be associated with early cerebral reinfarction. However, even though they included all types of acute ischemic stroke, paresis was not assessed, and low BP was associated with multiple early deaths from coronary heart events. Their study might have included more patients with heart diseases than our study because atrial fibrillation can cause cardioembolic infarction.^[26]^ However, the present study only included atherothrombotic infarction and demonstrated that approximately 20% patients without hypertension and nearly 10% patients with hypertension (BP: >140 and/or > 90) who were undergoing treatment exhibited low levels of ADL at hospital discharge (BI of 0–15, Fig. 2), although the effect of coronary heart events was not assessed. In addition, patients with severely low BP and OH were not excluded and were possibly included in the non-hypertensive group. Thus, our results highlight the impact of BP on inpatients after atherothrombotic infarction, showing that BP impacted a higher proportion of non-hypertensive inpatients than hypertensive inpatients, leading to severely low levels of ADL at hospital discharge. Further, our findings suggest that inpatients who did not have an optimal BP range that maintains cerebral blood flow in the sitting up posture (because of low BP or OH at baseline or induced by stroke) were included in the non-hypertensive group and had severely low levels of ADL at hospital discharge (Fig. 2). A previous study reviewed challenges related to the control of severely low BP using various methods.^[12]^ Memon et al^[13]^ reported a case of OH and demonstrated that OH management can be challenging despite multiple pharmaceutical and non-pharmaceutical options, with adverse effects of supine hypertension and variable responses, such as nausea, dizziness, and headaches, common to each therapy. In addition, a meta-analysis provided evidence of a strong association between OH and the subsequent risk of all-cause death, incident coronary heart disease, heart failure, and stroke, thereby supporting the findings of previous studies.^[27]^ As a result, we consider that these factors might have been present in the non-hypertensive group in this study and deteriorated BI improvement during rehabilitation.

This study has some limitations. First, our data were obtained based on the results of previous analyses in Asian populations, predominantly Japanese people, and none of the patients were of African or Caucasian descent; thus, the generalizability of our results to other ethnic groups is limited. Second, the tractus solitarius and ventrolateral medulla nuclei in the brain stem contain a network of respiratory, cardiovagal, and vasomotor neurons. Therefore, medullary autonomic disorders may cause OH, paroxysmal hypertension, and sleep apnea.^[10,28,29]^ However, we did not collect information on various stroke lesions in this study, which may have introduced a potential bias. Third, we did not consider the effect of poststroke cognitive impairment, which negatively impacts ADL.^[30]^ Fourth, the BI scoring system used in this study does not include an assessment of mortality, and this may have introduced bias in our findings.^[31]^ Fifth, a systematic protocol for recording BP was impossible, and we relied on measurements made during routine clinical practice at each hospital. Sixth, the BP readings were divided into hypertensive and non-hypertensive types, and individual readings were unavailable. Finally, a non-hypertensive condition negatively affected ADL recovery after atherothrombotic infarction, although the details remain unknown.

Nonetheless, our study also has several strengths. First, our study evaluated BP across a broad range, and no patient was excluded because of extremely low or high BP. Second, the combination of atherothrombotic infarction with assessment using the BI is excellent for the detection of differences in outcomes between hypertensive and non-hypertensive patients, unlike lacunar and cardioembolic infarctions; the former and latter are associated with generally milder and more severe ischemic strokes, respectively, than is atherothrombotic infarction. In addition, although significant differences were observed in scores for several BI activities, the BI at discharge was not significantly different; this has important implications in terms of daily life after stroke. To the best of our knowledge, this is the first study to use this combination with a focus on atherothrombotic infarction, BP, and individual ADL activities, and the results indicated the importance of precise observation based on individual BI activities. Third, precise assessments and analyses performed using BRS and assessment of the total BI and individual BI activities aided the observation of the impact of BP on ADL. Fourth, the JARD generated the dataset in this study, and the BRS and BI at hospital admission and discharge were assessed by clinicians who specialized in rehabilitation medicine and were well-versed with the BRS and BI tools and measurements. Finally, only patients with primary atherothrombotic infarction were included in the analyses, and no significant between-group differences were noted in terms of age and paresis, which can reportedly introduce bias.^[20]^

The findings of our study have important implications for the rehabilitation of patients with atherothrombotic infarction. The observed influence of BP on rehabilitation outcomes underscores the significance of managing hypertension during the recovery period. We need to consider each BI activity, as well as total BI, and consider a combination of methods to assess patients and types of stroke. Healthcare practitioners should consider BP control as an integral component of rehabilitation strategies to enhance ADL. These implications emphasize the need for further research on the effects of BP on various types of strokes and highlight the potential benefits of personalized rehabilitation plans based on patients’ BP profiles.

## 5. Conclusions

Our findings revealed more efficient ADL recovery and outcomes at hospital discharge in hypertensive patients than in non-hypertensive patients. This finding demonstrates the significant role of BP in ADL recovery and outcomes in patients with atherothrombotic infarction during rehabilitation. The data also suggest that a higher proportion of non-hypertensive patients are affected with BP than their hypertensive counterparts, probably because hypertensive patients have more advantages in maintaining optimal BP and cerebral blood flow during rehabilitation than those with non-hypertensive conditions such as normal BP or low BP undergoing treatment, which may also be relatable to other types of strokes. Therefore, the effects of low BP on ADL, various postures, and stroke types and lesions should be widely investigated in future studies, and analyses that include BI activities should be conducted to deepen our understanding of ADL and BP.

## Acknowledgments

The authors would like to thank the members of the Japan Association of Rehabilitation Database (JARD), who established and managed the database and gathered the data in collaboration with the investigators at the clinics and other study units. Without them, the performance of this study would not have been possible. The content is solely the responsibility of the authors and does not necessarily represent the official views of the JARD.

## Author contributions

**Data curation:** Naohito Saito.

**Formal analysis:** Naohito Saito.

**Methodology:** Naohito Saito, Tetsuo Ota.

**Project administration:** Naohito Saito.

**Resources:** Naohito Saito.

**Supervision:** Tetsuo Nishikawa, Tetsuo Ota.

**Writing – original draft:** Naohito Saito.

**Writing – review & editing:** Tetsuo Nishikawa, Tetsuo Ota.
